# Systematic Review of Genetic Factors in the Etiology of Esophageal Squamous Cell Carcinoma in African Populations

**DOI:** 10.3389/fgene.2019.00642

**Published:** 2019-08-02

**Authors:** Hannah Simba, Helena Kuivaniemi, Vittoria Lutje, Gerard Tromp, Vikash Sewram

**Affiliations:** ^1^African Cancer Institute, Division of Health Systems and Public Health, Department of Global Health, Faculty of Medicine and Health Sciences, Stellenbosch University, Cape Town, South Africa; ^2^Division of Molecular Biology and Human Genetics, Department of Biomedical Sciences, Faculty of Medicine and Health Sciences, Stellenbosch University, Cape Town, South Africa; ^3^Cochrane Infectious Diseases Group, Liverpool, United Kingdom; ^4^Bioinformatics Unit, South African Tuberculosis Bioinformatics Initiative, Stellenbosch University, Cape Town, South Africa; ^5^DST–NRF Centre of Excellence for Biomedical Tuberculosis Research, Stellenbosch University, Cape Town, South Africa; ^6^South African Medical Research Council Centre for Tuberculosis Research, Stellenbosch University, Cape Town, South Africa; ^7^Centre for Bioinformatics and Computational Biology, Stellenbosch University, Stellenbosch, South Africa

**Keywords:** esophageal squamous cell carcinoma, genetic association, somatic variant, germline mutation, sequence variants, systematic review, African populations

## Abstract

**Background:** Esophageal squamous cell carcinoma (ESCC), one of the most aggressive cancers, is endemic in Sub-Saharan Africa, constituting a major health burden. It has the most divergence in cancer incidence globally, with high prevalence reported in East Asia, Southern Europe, and in East and Southern Africa. Its etiology is multifactorial, with lifestyle, environmental, and genetic risk factors. Very little is known about the role of genetic factors in ESCC development and progression among African populations. The study aimed to systematically assess the evidence on genetic variants associated with ESCC in African populations.

**Methods:** We carried out a comprehensive search of all African published studies up to April 2019, using PubMed, Embase, Scopus, and African Index Medicus databases. Quality assessment and data extraction were carried out by two investigators. The strength of the associations was measured by odds ratios and 95% confidence intervals.

**Results:** Twenty-three genetic studies on ESCC in African populations were included in the systematic review. They were carried out on Black and admixed South African populations, as well as on Malawian, Sudanese, and Kenyan populations. Most studies were candidate gene studies and included DNA sequence variants in 58 different genes. Only one study carried out whole-exome sequencing of 59 ESCC patients. Sample sizes varied from 18 to 880 cases and 88 to 939 controls. Altogether, over 100 variants in 37 genes were part of 17 case-control genetic association studies to identify susceptibility loci for ESCC. In these studies, 25 variants in 20 genes were reported to have a statistically significant association. In addition, eight studies investigated changes in cancer tissues and identified somatic alterations in 17 genes and evidence of loss of heterozygosity, copy number variation, and microsatellite instability. Two genes were assessed for both genetic association and somatic mutation.

**Conclusions:** Comprehensive large-scale studies on the genetic basis of ESCC are still lacking in Africa. Sample sizes in existing studies are too small to draw definitive conclusions about ESCC etiology. Only a small number of African populations have been analyzed, and replication and validation studies are missing. The genetic etiology of ESCC in Africa is, therefore, still poorly defined.

## Introduction

Esophageal cancer is an aggressive and fatal cancer of the 18digestive tract. It accounts for an estimated 455,800 new cases and 400,200 deaths per year globally, making it the eighth most common cancer in the world (Murphy et al., 2017). The malignant tumors are characterized by two major subtypes: esophageal squamous cell carcinoma (ESCC), which is the more common type and contributes 90%, and esophageal adenocarcinoma (EAC) (Kaz and Grady, 2014; Abnet et al., 2017). ESCC presents with poor prognosis and low survival rate (<5%) in low resource settings (Yazbeck et al., 2016; Murphy et al., 2017). The asymptomatic development of ESCC results in diagnosis at late stage for patients and is characterized by dysphagia. At this stage, treatment is limited to palliative care.

ESCC is endemic in specific geographic locations worldwide and has the most divergence in cancer incidence globally, with high prevalence reported in East Asia, Southern Europe, as well as in Eastern and Southern Africa (Abnet et al., 2017). This peculiar distribution draws questions on the specificity of certain risk factors to particular populations. The African ESCC corridor, which includes Ethiopia, Rwanda, Burundi, Malawi, Kenya, Uganda, Tanzania, and South Africa, is an ESCC hotspot region (Munishi et al., 2015; Schaafsma et al., 2015). It has also been reported that in Sub-Saharan Africa, ESCC develops in younger patients than in other regions (Kayamba et al., 2015).

The etiology of esophageal carcinoma is multifactorial. The risk factors reported worldwide comprise several lifestyle and environmental and genetic factors (Pink et al., 2011; Sewram et al., 2014; Chen et al., 2015; Sewram et al., 2016; Huang and Yu, 2018). Growing evidence supports the hypothesis that genomic alterations and epigenetic modifications contribute to tumor development (Baba et al., 2017). ESCC has both an inherited and cellular genetic basis (Abnet et al., 2017; Coleman et al., 2018). Familial syndromes associated with increased risk of malignancy include tylosis and Fanconi anemia (Abnet et al., 2017). The majority of genetic studies on ESCC have been case-control association studies analyzing single-nucleotide polymorphisms (SNPs) in various candidate genes. However, the reproducibility of these studies has been low. Some of the more common SNPs associated with ESCC have been identified in the aldehyde dehydrogenase 2 family gene (*ALDH2*) and an acetaldehyde dehydrogenase gene *(ADH1B)* (Abnet et al., 2017). Variants in these genes have been shown to increase susceptibility to ESCC development, and they are also associated with alcohol consumption (Abnet et al., 2017). Two meta-analyses published in 2018 reported associations between the genes *MTHFR* and *GSTT1* and esophageal cancer development (He et al., 2018; Kumar and Rai, 2018). However, the meta-analyses were done on predominantly Asian and Western populations. In recent years, the focus of ESCC research in the Western and Asian countries has shifted from candidate gene studies to genome-wide association studies (GWAS) and whole-exome sequencing (WES) to identify variants associated with ESCC. Combined analysis of different study designs has provided a better understanding of ESCC etiology in Asian populations (Abnet et al., 2017). Genes with variants implicated in the development of ESCC in these populations include phospholipase c epsilon 1 *(PLCE1)*, caspase 8 *(CAP8)*, tumor protein 53 *(TP53)*, and human leukocyte antigen *(HLA)* (Abnet et al., 2017).

The genetic etiology of ESCC in Africa is not well understood, since there have been very few studies on ESCC in African populations. This is in part due to the unavailability of adequate research infrastructure. A lack of comprehensive assessment and validation of existing evidence through systematic reviews has also contributed to this knowledge gap. A number of small studies on African populations have yielded varied associations between genetic variants and ESCC. There is, therefore, a need to systematically assess the current evidence in order to map out the contribution of genetic factors in the development of ESCC in African populations using critically appraised data.

The aim of the current systematic review was to assess all genetic (cross-sectional, case-control, and cohort) studies reporting on germline and somatic variants where risk factor estimates were calculated. This was achieved through the following: 1) critical appraisal of African literature on association of genetic factors to ESCC development; 2) comprehensive analysis of genetic (germline and somatic) variants in the reported studies; 3) data synthesis through pooled analysis, if feasible; and 4) comparison of genetic variants identified in African populations to those reported in other geographic regions.

## Materials and Methods

We followed the Preferred Reporting Items for Systematic Reviews and Meta-Analyses guidelines (PRISMA) (Little et al., 2009). However, because PRISMA is not a quality assessment tool, other instruments were used to assess quality control.

### Data Sources and Search Strategy

We carried out a literature search on all published African ESCC studies up to April 2019. We developed a comprehensive set of search terms subjectively and iteratively. We searched the following electronic bibliographic databases without time or language limits: Medline (PubMed), Embase (OViD), Scopus, African Index Medicus, and Africa-wide information (EbsCOHost). We also checked the reference lists of potentially relevant articles for additional citations and used the “related citations” search key in PubMed to identify similar papers.

We checked Medline (PubMed) to identify controlled vocabulary (MeSH) terms related to esophageal cancer and also identified text keywords based on our knowledge of the field (**Table 1**). Medline search terms were modified for other electronic databases to conform to their search functions.

**Table 1:** Medline (PubMed) search strategy to identify published African ESCC literature.

#1	Search cancer or carcinoma or neoplasm* Field: Title/Abstract
#2	Search Esophageal or oesophageal Field: Title/Abstract
#3	#1 and #2
#4	Search “Esophageal cancer” Field: Title/Abstract
#5	Search “oesophageal cancer” or “oesophageal neoplasm*” Field: Title/Abstract
#6	Search “Esophageal Neoplasms”[Mesh]
#7	Search “Esophageal Neoplasms” Field: Title/Abstract
#8	Search “Esophageal squamous cell carcinoma” or “oesophageal squamous cell carcinoma” or ESCC Field: Title/Abstract
#9	Search ((((#3) OR #4) OR #5) OR #6) OR #7 OR #8
#10	Search “Africa”[Mesh]
#11	Search algeria OR angola OR benin OR botswana OR burkina faso OR burundi OR cameroon OR cape verde OR central african republic OR chad OR comoros OR congo OR “Democratic Republic of Congo” OR DRC OR djibouti OR equatorial guinea OR egypt OR eritrea OR ethiopia OR gabon OR gambia OR ghana OR guinea OR bissau OR ivory coast OR (Côte d’ Ivoire) OR jamahiriya OR kenya OR lesotho OR liberia OR Libya OR madagascar OR malawi OR mali OR mauritania OR mauritius OR mayotte OR morocco OR mozambique OR namibia OR niger OR nigeria OR principe OR reunion OR rwanda OR “Sao Tome” OR senegal OR seychelles OR “Sierra Leone” OR somalia OR “South Africa” OR st helena OR sudan OR swaziland OR tanzania OR togo OR tunisia OR uganda OR zaire OR zambia OR zimbabwe OR “Central Africa” OR “West Africa” OR “East Africa” OR “Southern Africa” OR “South Africa” Field: Title/Abstract
#12	Search (#10) or #11
#13	Search (#9) AND #12

**Figure 1:**
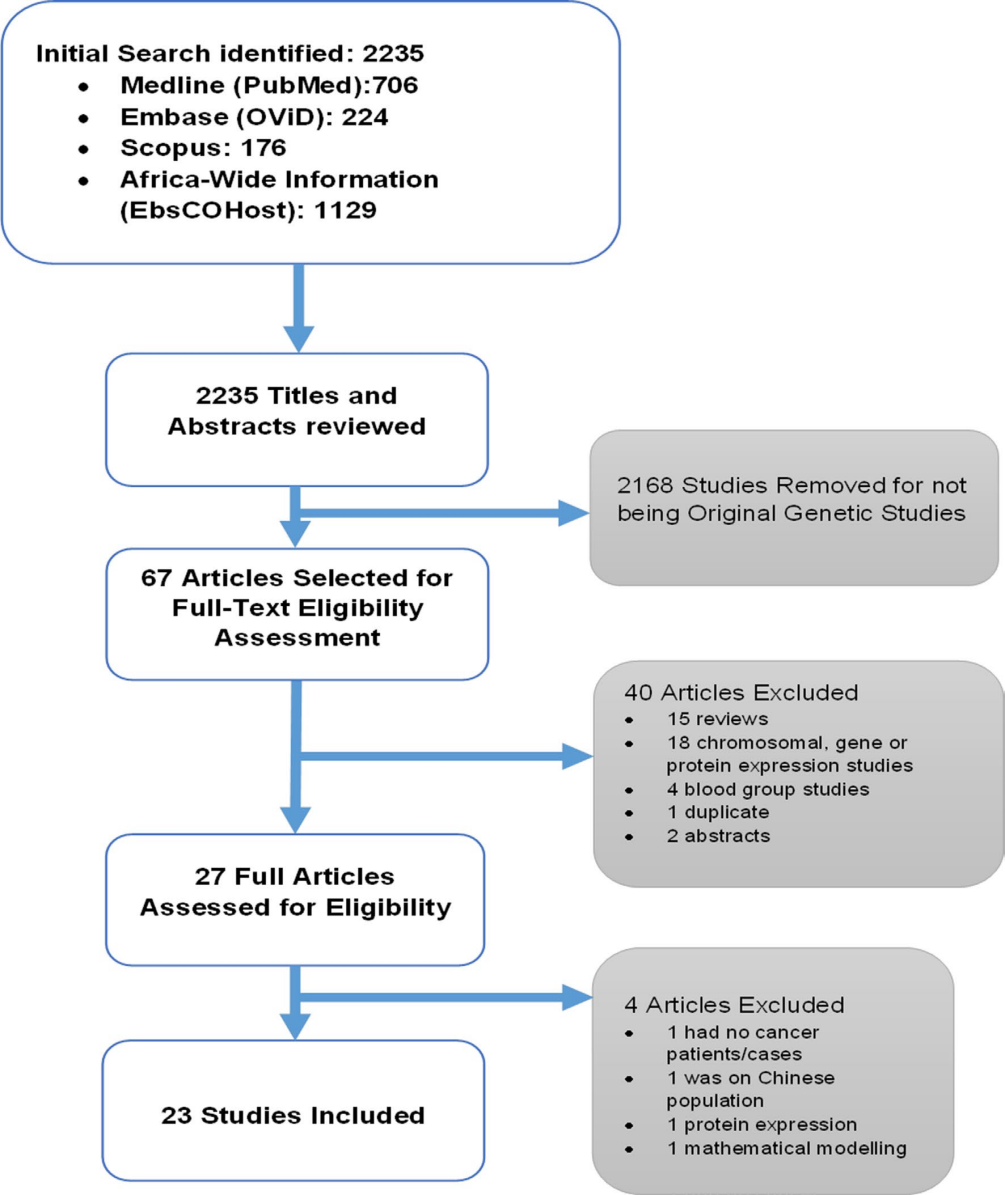
Outline of the systematic review.

Screening for eligible studies was carried out by two authors (HS and HK). First, the two authors read the titles and abstracts independently and then met to finalize an initial list. Full articles of the studies selected based on the initial screening were read and assessed for inclusion to the systematic review. **Figure 1** shows the outline for selection of eligible studies.

### Quality Control and Data Extraction

Quality of the methodology used in the published studies was assessed using a quality assessment tool adapted from the STrengthening the REporting of Genetic Association studies (STREGA) statement (Little et al., 2009). The quality assessment for genetic association studies to identify ESCC susceptibility loci included reporting on power calculations, detailed population characteristics for cases, description of ESCC diagnosis, screening of cases and controls, reporting a measure of association using odds ratios, adjustment of population stratification, assessment of genotyping error, reporting the Hardy–Weinberg equilibrium, correction for multiple testing, and reporting of National Center for Biotechnology Information (NCBI) rs numbers for variants (**Table S1**).

For somatic mutation studies, quality assessment included the following: description of ESCC diagnosis, reporting of tissues used [cancerous (Ca) and normal neighboring tissue (NET)], detailed population characteristics, variant classification and type, confirmation of variants identified, reporting of amino acid change, and use of pathogenicity scoring (**Table S2**).

Data extraction was carried out by two authors (HS and HK) using data extraction forms. Two separate extraction forms were prepared for the germline (genetic susceptibility) and somatic mutation studies. The data extraction form for the genetic susceptibility studies included the following: description of the population (age, sex, sample size, smoking, and alcohol use for cases and controls separately), genotyping method, statistical analysis test, minor allele frequency (MAF), genotype frequency, haplotype frequency, and environmental association frequency. The somatic mutation study extraction form had the same variables excluding gene–environment interaction frequency and haplotype frequency.

The South African Admixed Population is reported as mixed ancestry in the tables according to how it was reported in the articles.

### Data Analysis

A meta-analysis could not be performed as there were only two SNPs analyzed in more than one study and even those were analyzed in only two independent studies. For a meta-analysis to be carried out, SNPs have to be assessed in at least three separate case-control studies. *TP53* in the somatic variant studies was analyzed in four separate studies, but two of the studies had cases only with no controls, and the remaining two assessed different parts of the gene. The results of this systematic review will, therefore, be reported in a descriptive manner.

We were able to find rs numbers for most of the variants even if the authors of the original studies did not report them and have included them in the tables of this systematic review. We used the canonical SNP identifier (rs number) and dbSNP (version 152; April 2019) database at NCBI (https://www.ncbi.nlm.nih.gov/snp/) for this. We also determined the locus positions of the microsatellite markers reported in a study by Naidoo et al. (2005) using the primer-BLAST database at NCBI (https://www-ncbi-nlm-nih-gov.ez.sun.ac.za/tools/primer-blast).

To determine the linkage disequilibrium (LD) measures between the SNPs reported in the same genes, we obtained the imputed data set from the Thousand Genomes project (1000 Genomes Release Phase 3 2013-05-02) and used bcftools to extract all individuals from African populations, not including African Americans, and the 77 SNPs discussed here using all synonyms (alternative rs IDs) for SNPs (Auton et al., 2015). We obtained a dataset of 504 individuals and 67 SNPs. We computed all pair-wise r^2^-values using PLINK (v1.09) (Danecek et al., 2011; Chang et al., 2015).

## Results

### Systematic Review Outline

The selection process for all the included studies is shown in **Figure 1**. The initial database search identified 2,235 articles. Titles and abstracts of these articles were reviewed, and 2,168 studies were removed for not being original genetic studies. The 67 articles that remained were selected for full-text eligibility assessment. This process resulted in the removal of 40 articles: 15 review articles, 18 chromosomal, gene or protein expression studies, 4 blood group studies, 1 duplicate, and 2 abstracts. A total of 27 full articles were then assessed for eligibility, and four articles were removed for not meeting the criteria, as follows: one study had no cancer patients/cases (Adams et al., 2003), one focused on the Chinese population (Li et al., 2016), while one focused on protein expression (Jaskiewicz and De Groot, 1994; Huang and Yu, 2018), and the other was a mathematical model study (Uys and Van Helden, 2003). In the end, 23 studies were included and analyzed in the systematic review.

### Study Characteristics

The characteristics of all the genetic susceptibility and somatic variant studies included are shown in **Tables 2** and **3**, respectively. The 23 studies included in the study were published between 1990 and 2019. There were 17 genetic susceptibility and eight somatic variant studies. Two studies reported on both genetic susceptibility and somatic variants.

**Table 2:** Characteristics of genetic susceptibility studies for ESCC in African populations.

Study (PMID)	Location	Year	Population	Age, y (SD)		Sample size		Sex, cases n (%)		Sex, ctrl n (%)		Clinical assessment		Analysis method	Smoking n (%)		Alcohol n (%)	
				Cases	Ctrl	Cases	Ctrl	Male	Female	Male	Female	Cases	Ctrl		Cases	Ctrl	Cases	Ctrl
Bye et al., 2011 (21926110)	South Africa	2011	Black	59.8 (11.3)	–	358	477	182 (50.8)	176 (49.2)	–	–	Histology	–	TaqMan Assay	228 (63.7)	–	228 (63.7)	–
			Mixed ancestry	60.5 (10.6)	–	201	427	131 (65.2)	70 (34.8)	–	–	Histology	–	TaqMan Assay	189 (94.1)	–	163 (81.1)	–
Bye et al., 2012 (22865593)	South Africa	2012	Black	59.8 (11.3)	48.8 (16.7)	407	849	199 (48.9)	208 (51.1)	335 (39.5)	511 (60.2)	Histology	–	TaqMan Assay and KASP	242 (59.5)	333 (39.2)	253 (62.2)	452 (53.2)
			Mixed ancestry	60.6 (10.6)	46.7 (16.8)	257	860	165 (64.2)	91 (35.4)	309 (35.9)	551 (64.1)	Histology	–	TaqMan Assay and KASP	240 (93.4)	597 (69.4)	212 (82.5)	419 (48.7)
Chelule et al., 2006 (17264406)	South Africa	2006	Black	18–74^1^	18–74	70	261	–	–	–	–	Histology	–	PCR-RFLP	–	–	–	–
Chen et al., 2019 (30753320)	South Africa	2019	Black^7^	60.2 (11.3)	48.9 (16.8)	591	852	284 (48.1)	307 (51.9)	342 (40.1)	507 (59.5)	Histology	–	TaqMan Assay	364 (61.6)	338 (39.7)	370 (62.6)	458 (53.7)
			Black^8^	58.2 (10.2)	50.0 (15.5)	880	939	545 (61.9)	332 (37.7)	240 (25.6)	698 (74.3)	Histology		iPLEX and TaqMan Assays	598 (68.0)	333 (35.5)	473 (53.8)	633 (67.4)
Dandara et al., 2005 (15978331)	South Africa	2005	Black	–	–	142	178	–	–	–	–	Histology	–	PCR-RFLP	179	162	171	160
			Mixed ancestry	–	–	99	94					Histology		PCR-RFLP				
Dandara et al., 2006 (16272171)	South Africa	2006	Black	61.23	61.85	145	194	85 (59)	60 (41)	111 (57)	83 (43)	Histology	–	PCR-RFLP	95 (65)	123 (63)	98 (68)	127 (65)
			Mixed ancestry	61.49	69.53	100	94	78 (78)	22 (22)	45 (48)	49 (52)	Histology	–	PCR-RFLP	93 (93)	74 (79)	73 (73)	45 (48)
Dietzsch et al., 2003 (12925954)	South Africa	2003	Black and mixed ancestry	59.6	58.7	58^2^	226	44	14	167	59	–	–	PCR and PAGE				
Eltahir et al., 2012 (23053979)	Sudan	2012				18	235					Histology		PCR-RFLP				
Li et al., 2005 (15899651)	South Africa	2005	Black and mixed ancestry	61.1 (10.5)	65.7 (10.2)	189	198	–	–	–	–	Histology	–	PCR-SSCP and DNA sequencing	144 (76)	122 (62)	133 (70)	114 (58)
Li et al., 2008 (18254707)	South Africa	2008	Black^3^	–	–	142	178	–	–	–	–	Histology	–	PCR- RLFP	179	162	71	160
			Mixed^3^ ancestry			101	100					Histology		PCR-RFLP				
Li et al., 2010 (20540773)	South Africa	2010	Black^3^	61.23	61.85	145	194	85 (59)	60 (41)	111 (57)	83 (43)	Histology	–	PCR-RFLP	95 (65)	123 (63)	98 (68)	127 (65)
			Mixed^3^ ancestry	61.49	69.53	100	94	78 (78)	22 (22)	45 (48)	49 (52)	Histology	–	PCR- RFLP	93 (93)	74 (79)	73 (73)	45 (48)
Matejcic et al., 2011 (22216261)	South Africa	2011	Black	–	–	330	479	–	–	–	–	Histology	–	TaqMan assay and gel electrophoresis	210	–	204	–
			Mixed ancestry	–	–	232	428	–	–	–	–	Histology	–	TaqMan assay and gel electrophoresis	216	–	189	–
Matejcic et al., 2015 (26447020)	South Africa	2015	Black	59.6 (10.7)	56.7 (15.0)	463	480	229 (49)	234 (51)	235 (49)	245 (51)	Histology	–	TaqMan assay	280 (60)	222 (46)	286 (62)	278 (58)
			Mixed ancestry	60.7 (10.3)	57.7 (14.3)	269	288	177 (66)	92 (34)	178 (62)	110 (38)	Histology	–	TaqMan Assay	250 (93)	226 (78)	215 (80)	172 (60)
Strickland et al., 2012 (21901748)	South Africa	2012	Black	59/66^4^	–	96	88	48	48	–	–	Histology	Brush biopsy	HEX SSCP and DNA sequencing	58	–	58	–
Vogelsang et al., 2012 (22623965)	South Africa	2012	Black	59.8 (11.3)	56.1 (16.2)	345^5^	344	166 (48.1)	179 (51.9)	120 (34.9)	224 (65.1)	Histology	–	Allele-specific quantitative PCR	209 (60.6)	117 (34.0)	160 (46.4)	92 (26.7)
			Mixed ancestry	60.7 (10.2)	56.8 (16.5)	205^6^	266	136 (66.3)	69 (33.7)	82 (30.8)	184 (69.2)	Histology	–	Allele-specific quantitative PCR	189 (92.2)	162 (60.9)	118 (57.6)	38 (14.3)
Vos et al., 2003 (12550754)	South Africa	2003	Black	57 (11)	57 (11)	74	118	–	–	–	–	Histology	–	SSCP and DNA sequencing	–	–	–	–
Zaahl et al., 2005 (15860357)	South Africa	2005	Mixed ancestry	–	–	105	110	82	23	43	67	Histology	–	SSCP and DNA sequencing	–	–	–	–

^1^Only range of age was reported for the combined group of cases and controls.

^2^57 had ESCC.

^3^Same population as in Dandara et al. (2005) study.

^4^59+/–13 for male (n = 48) and 66+/– (n = 48) for female patients.

^5^326 had ESCC.

^6^182 had ESCC.

^7^Western and Eastern Cape Province Black Population.

^8^Gauteng Province Black Population.

Ctrl, controls; ESCC, esophageal squamous cell carcinoma; HEX, heteroduplex; KASP, competitive allele specific PCR; PAGE, polyacrylamide gel electrophoresis; PCR, polymerase chain reaction; RFLP, restriction fragment length polymorphism; SD, Standard deviation; SSCP, single-strand conformation polymorphism.

**Table 3:** Characteristics of studies on somatic changes in ESCC in African populations.

Study (PMID)	Country	Year	Population	Sample size			Age, y (SD)	Sex n (%)		Clinical assessment		Analysis method	Smoking n (%)	Alcohol n (%)
				Ca	NET	Blood	Cases	Male	Female	Ca	NET			
Dietzsch and Parker, 2002 (12435113)	South Africa	2002	Black	33	33	–	57.4	23 (70)	10 (30)	Histology	–	PCR and DNA sequencing analysis	–	–
Dietzsch et al., 2003 (12925954)	South Africa	2003	Black and mixed ancestry	58^1^	58	–	59.6	29 (67)	14 (33)	–	–	PCR and PAGE	–	–
Gamieldien et al.,1998 (9808520)	South Africa	1998	Black	76	9	50	57 (11)	49 (65)	27 (35)	Histology	Histology	PCR and HEX-SSCP	–	–
Liu et al., 2016 (29148985)	Malawi	2016	Malawian	59	–	59	56	27 (45.8)	31 (52.5)	Histology	-	WES	24 (40.7)	14 (23.7)
Naidoo et al., 2005 (15735161)	South Africa	2005	South African	100	100	–	56	53 (54)	45 (46)	Histology	Histology	PCR	–	–
Patel et al., 2011 (22040862)	Kenya	2011	Kenyan	28	–	–	56.03 (12.30)	13 (46)	15 (54)	–	–	PCR and DNA sequencing	6 (21)	10 (36)
Victor et al., 1990 (2199031)	South Africa	1990	Black and mixed ancestry	27	–	–	–	–	–	–	–	PCR and dot blot hybridization	–	–
Vos et al., 2003 (12550754)	South Africa	2003	South African	74	–	37	–	–	–	Histology	–	SSCP and DNA sequencing	–	–

Ca, cancer tissue; HEX-SSCP, heteroduplex single-strand conformation polymorphism; NET, neighboring tissue; PAGE, polyacrylamide gel electrophoresis; PCR, polymerase chain reaction; WES, whole exome sequencing.

^1^57 had ESCC and 1 had adenocarcinoma.

#### Genetic Susceptibility Studies

The 17 genetic susceptibility studies (**Table 2**) were all case-control studies (Dietzsch et al., 2003; Vos et al., 2003; Dandara et al., 2005; Li et al., 2005; Zaahl et al., 2005; Chelule et al., 2006; Dandara et al., 2006; Li et al., 2008; Li et al., 2010; Bye et al., 2011; Matejcic et al., 2011; Bye et al., 2012; Eltahir et al., 2012; Strickland et al., 2012; Vogelsang et al., 2012; Matejcic et al., 2015; Chen et al., 2019) published between 2003 and 2019. Sixteen articles reported on the South African population and one article on the Sudanese population. The majority (13/17; 76%) of the studies reported on the main subject characteristics (ethnicity, sex, age, and type of clinical assessment). Sample sizes for ESCC patients ranged from 18 to 880 with six of the studies having over 200 patient samples. Sample sizes for controls ranged from 88 to 939 with nine of the studies having over 200 control samples. It is difficult to estimate the total number of patients analyzed in these 17 studies, since it appears that the same authors used the same sample set for different SNPs in different publications. Our assessment showed that Bye et al. (2011) and Bye et al. (2012) used the same participants. In addition, studies by Li et al. (2005)and Li et al. (2008) used the same participants as Dandara et al. (2005). The remaining 12 studies do not seem to have any obvious sample overlap.

Altogether, 16 out of 17 studies clinically assessed for ESCC through histology. None of the studies clinically assessed controls for ESCC with the exception of one study (Strickland et al., 2012), which assessed controls using a brush biopsy. Nine studies reported on smoking and alcohol consumption status for all participants (Dandara et al., 2005; Li et al., 2005; Dandara et al., 2006; Li et al., 2008 Li et al., 2010; Bye et al., 2012; Vogelsang et al., 2012; Matejcic et al., 2015; Chen et al., 2019), while three (Bye et al., 2011; Matejcic et al., 2011; Strickland et al., 2012) reported those risk factors for only the ESCC patients.

The Hardy–Weinberg equilibrium deviation was assessed in 11 (65%) studies; however, only six (35%) of the studies reported power calculations, and three (18%) studies reported the evaluation of a genotyping error. Detailed characteristics of the study population were reported in 12 of the studies for cases and 10 for controls. Correction for multiple testing was reported in only seven (41%) studies. NCBI rs numbers were reported in eight (47%) studies. Our quality assessment scoring had 11 items (**Table S1**), and each item had a weight of 1 point; therefore, total maximum quality score was 11. Overall, only seven of the 17 (41%) studies scored half or above half (5.5). The highest score was 9 (Vogelsang et al., 2012; Chen et al., 2019), and the lowest score was 1 (Vos et al., 2003; Zaahl et al., 2005).

#### Somatic Variant Studies

Somatic variant studies (**Table 3**) constituted of eight studies published between 1990 and 2016 (Victor et al., 1990; Gamieldien et al., 1998; Dietzsch and Parker, 2002; Dietzsch et al., 2003; Vos et al., 2003; Naidoo et al., 2005; Patel et al., 2011; Liu et al., 2016). A total of 455 patients were assessed, with the control group comprising 200 NET and 146 blood samples. Of the 455 patient samples, one was reported to be an adenocarcinoma from one study; therefore, the exact ESCC patient population was 454. The study populations were from South Africa, Kenya, and Malawi.

Clinical diagnosis of ESCC was determined by histology in five (75%) studies, and the remaining three did not report on how clinical assessment was done. Four (50%) studies reported using both cancer tissue and NET for assessment. Three of these studies had an equal number of cancer tissue and NET samples. Two (25%) studies did not have any control samples, and the remaining two (25%) studies collected blood samples only as controls. Only two studies reported on smoking and alcohol consumption status. On patient characteristics, age and sex were reported in six (75%) of the studies. Variant classification and type were reported in all of the studies, but confirmation of results was reported in only two studies. No studies used pathogenicity scoring. Amino acid change was also reported in only two of the studies. Our quality assessment score had seven items (**Table S2**), and each item had a weight of 1 point; therefore, total maximum score for the quality assessment was 7. Overall, six of the eight (75%) studies scored half or above half (3.5). The highest score was 6 (Gamieldien et al., 1998), and the lowest score was 0 (Victor et al., 1990).

### Description of Genes Studied

A total of 58 genes were investigated in the 23 studies, which were selected for the systematic review, with 37 genes studied in the genetic susceptibility studies and 23 in the somatic variant studies. Two genes were investigated in both studies. In addition, the somatic studies investigated six genetic loci without specific gene names. A summary of SNPs analyzed in the genetic susceptibility studies is shown in **Table 4**. Over 100 SNPs were analyzed, and 25 SNPs were reported to be associated with ESCC (four SNPs using p values only, and 21 SNPs using p values and odds ratios). The 25 SNPs were in 20 genes: *ADH1B, ADH3, ALDH2, AR, CASP8, CHEK2, CP, CYP2E1, CYP3A5, GSTT2B, MGMT, MLH3, MSH3, NAT2, PTGS2 (also known as COX-2), PLCE1, PMS1, RUNX1, SLC11A1, and TP53*. The associations with all 25 SNPs were identified in South African populations, while none were found in the Sudanese population.

**Table 4:** Summary of studies investigating genetic susceptibility of ESCC in African populations.

Gene	Variant (rs number)	Study (PMID)	Population	ESCC		Controls		Effect allele	Findings and Comments^2^
								n	MAF	n	MAF		
*ADH1B*	rs1229984 (Arg48His)	Bye et al., 2011 (21926110)	Black South African	358	0	477	0		Not informative
		Bye et al., 2011 (21926110)	Mixed ancestry South African	201	0.054	427	0.098	A	OR = 0.52 (0.32–0.86) p = 0.009
*ADH2*	ADH2*1/*2/*3	Li et al., 2008 (18254707)	Black South African	142	0.01	174	0.01		Not informative
		Li et al., 2008 (18254707)	Mixed ancestry South African	96	0.03	94	0.03		Not informative
*ADH3*	ADH3*1/*2	Li et al., 2008 (18254707)	Black South African	141	0.46	174	0.32		NS
		Li et al., 2008 (18254707)	Mixed ancestry South African	96	0.38	94	0.31	*2	OR = 1.80; p = 0.0004
*ADH7*	rs1573496 (Gly92Ala)	Bye et al., 2011 (21926110)	Black South African	358	0	477	0.001		Not informative
		Bye et al., 2011 (21926110)	Mixed ancestry South African	201	0.014	427	0.02		NS
*ALDH2*	rs671 (Glu504Lys)	Bye et al., 2011 (21926110)	Black South African	358	0	477	0		Not informative
		Bye et al., 2011 (21926110)	Mixed ancestry South African	201	0	427	0		Not informative
	rs441 (-261 C/T)	Bye et al., 2011 (21926110)	Black South African	358	0.154	477	0.145		NS
		Bye et al., 2011	Mixed ancestry South African	201	0.18	427	0.194		NS
	rs886205 (+82 A/G)	Bye et al., 2011 (21926110)	Black South African	358	0.247	477	0.252		NS
		Bye et al., 2011 (21926110)	Mixed ancestry South African	201	0.402	427	0.489	G	OR = 0.70 (0.55–0.89); p = 0.004
	ALDH2*1/*2	Li et al., 2008 (18254707)	Black South African	142	0.10	174	0.04	*2	OR = 2.35; p = 0.008
		Li et al., 2008 (18254707)	Mixed ancestry South African	101	0.03	1004	0.04		Not informative
	rs4767364 (A/G)	Chen et al., 2019 (30753320)	Black South African^5^	880	0.12	939	0.11		NS
*ALS2CR12*	rs13016963 (G/A)	Chen et al., 2019 (30753320)	Black South African^4^	591	0.35	852	0.35		NS
		Chen et al., 2019 (30753320)	Black South African^5^	880	0.39	939	0.38		NS
	rs10201587 (A/G)	Chen et al., 2019 (30753320)	Black South African^5^	880	0.38	939	0.39		NS
*AR*	CAG-repeat in exon 1	Dietzsch et al., 2003 (12925954)	Black South African males	29		109			NS
		Dietzsch et al., 2003 (12925954)	Mixed ancestry South African males	15		58			NS
	GGC-repeat in exon 1	Dietzsch et al., 2003 (12925954)	Black South African males	29		109		(GGC)_≤16_	OR = 2.7 (1.14–6.36); p = 0.018
		Dietzsch et al., 2003 (12925954)	Mixed ancestry South African males	15		58			NS
*ATP1B2/TP53*	rs1642764 (C/T)	Chen et al., 2019 (30753320)	Black South African^4^	591	0.21	852	0.20		NS
		Chen et al., 2019 (30753320)	Black South African^5^	880	0.18	939	0.18		NS
	rs1641511 (A/G)	Chen et al., 2019 (30753320)	Black South African^5^	880	0.39	939	0.42		NS
*C20orf54*	rs13042395	Bye et al., 2012 (22865593)	Black South African	407	0.002	849	0.005		Not informative
		Bye et al., 2012 (22865593)	Mixed ancestry South African	257	0.067	860	0.068		NS
*CASP8*	rs1045485 (Asp302His)	Bye et al., 2011 (21926110)	Black South African	358	0.154	477	0.152		NS
		Bye et al., 2011 (21926110)	Mixed ancestry South African	201	0.169	427	0.126	C	OR = 1.42 (1.01–1.98); p = 0.040
	rs3834129 (-652 6N ins/del)	Bye et al., 2011 (21926110)	Black South African	358	0.518	477	0.502		NS
		Bye et al., 2011 (21926110)	Mixed ancestry South African	201	0.385	427	0.386		NS
	rs10931936 (C/T)	Chen et al., 2019 (30753320)	Black South African^4^	591	0.19	852	0.20		NS
		Chen et al., 2019 (30753320)	Black South African^5^	880	0.22	939	0.20		NS
*CHEK2*	rs4822983 (C/T)	Chen et al., 2019 (30753320)	Black South African^4^	591	0.46	852	0.39	T	OR = 1.32 (1.12–1.56); p = 0.001
		Chen et al., 2019 (30753320)	Black South African^5^	880	0.43	939	0.42		NS
	rs1033667 (C/T)	Chen et al., 2019 (30753320)	Black South African^4^	591	0.44	852	0.38	T	OR = 1.30 (1.10–1.53) P = 0.002
		Chen et al., 2019 (30753320)	Black South African^5^	880	0.42	939	0.39		NS
*CP*	rs34053109 (C/G)	Strickland et al., 2012 (21901748)	Black South African	84	0	85	0.01		Not informative
	rs17838834 (T/C)	Strickland et al., 2012 (21901748)	Black South African	90	0.33	85	0.23		NS
	rs701749 (C/T)	Strickland et al., 2012 (21901748)	Black South African	79	0.01	78	0.02		Not informative
	rs17838833 (delT)	Strickland et al., 2012 (21901748)	Black South African	79	0.01	78	0		Not informative
	rs17838832 (T/C)	Strickland et al., 2012 (21901748)	Black South African	80	0.33	78	0.3		NS
	rs34334174 (C/T)	Strickland et al., 2012 (21901748)	Black South African	80	0.14	78	0.08		NS
	5’UTR-308G/A	Strickland et al., 2012 (21901748)	Black South African	52	0.05	64	0	A	p = 0.012; sample size very small
	rs17838831 (A/G)	Strickland et al., 2012 (21901748)	Black South African	53	0.21	64	0.22		NS
	rs138512757 (Thr83)	Strickland et al., 2012 (21901748)	Black South African	92	0.02	84	0.01		Not informative
	rs35438054 (Val223)	Strickland et al., 2012 (21901748)	Black South African	95	0.01	85	0.01		Not informative
	rs797045480 (Val246Ala)	Strickland et al., 2012 (21901748)	Black South African	95	0.01	85	0		Not informative
	rs34067682 (IVS4-14C/T)	Strickland et al., 2012 (21901748)	Black South African	84	0.12	83	0.12		NS
	rs34624984 (Arg367Cys)	Strickland et al., 2012 (21901748)	Black South African	94	0.02	86	0.01		Not informative
	rs34237139 (Tyr425)	Strickland et al., 2012 (21901748)	Black South African	91	0.01	87	0		Not informative
	rs35272481 (IVS7+9T/C)	Strickland et al., 2012 (21901748)	Black South African	91	0.01	87	0		Not informative
	rs701753 (D544E)	Strickland et al., 2012 (21901748)	Black South African	95	0.23	81	0.27		NS
	rs147192657 (Gly633 T/C)	Strickland et al., 2012 (21901748)	Black South African	88	0.07	84	0	C	p = 0.0004
	rs16861582 (IVS15-12T/C)	Strickland et al., 2012 (21901748)	Black South African	93	0,44	88	0.41		NS
*CYP2E1*	CYP2E1*1 (c1)/CYP2E1*5 (c2)	Chelule et al., 2006 (17264406)	Black South African	30	0.04	331	0.06		Limited power
	-1053C/T	Li et al., 2005 (15899651)	Black and Mixed ancestry South African	189	0.01	198	0.02		NS
	-1293G/A	Li et al., 2005 (15899651)	Black and Mixed ancestry South African	189	0.01	198	0.03		NS
	7632T/A	Li et al., 2005 (15899651)	Black and Mixed ancestry South African	189	0.18	198	0.07	A	OR = 5.90 (3.25–10.7); p = 0.001 for genotype distribution
*CYP3A5*	CYP3A5*1	Dandara et al., 2005 (15978331)	Black South African	142	0.627	178	0.638		NS
		Dandara et al., 2005 (15978331)	Mixed ancestry South African	99	0.384	94	0.287		NS
	CYP3A5*3 (6986A/G)	Dandara et al., 2005 (15978331)	Black South African	142	0.155	178	0.138		NS
		Dandara et al., 2005 (15978331)	Mixed ancestry South African	99	0.475	94	0.590	G	OR = 0.60 (0.39–0.94); p = 0.025
	CYP3A5*6 (1490G/A)	Dandara et al., 2005 (15978331)	Black South African	142	0.190	178	0.213		NS
		Dandara et al., 2005 (15978331)	Mixed ancestry South African	99	0.136	94	0.122		NS
	CYP3A5*7 (27131-32insT; frameshift)	Dandara et al., 2005 (15978331)	Black South African	142	0.028	178	0.011		NS
		Dandara et al., 2005 (15978331)	Mixed ancestry South African	99	0.005	94	0		Not informative
	CYP3A5 all variants	Dandara et al., 2005 (15978331)	Black South African	142	0.373	178	0.441		NS
		Dandara et al., 2005 (15978331)	Mixed ancestry South African	99	0.616	94	0.713		OR = 0.65 (0.42–0.99); p = 0.045
*FAS*	rs1800682 (-670 G > A)	Bye et al., 2011 (21926110)	Black South African	358	0.219	477	0.225		NS
		Bye et al., 2011 (21926110)	Mixed ancestry South African	201	0.356	427	0.406		NS
	rs2234767 (-1377 G > A)	Bye et al., 2011 (21926110)	Black South African	358	0.096	477	0.072		NS
		Bye et al., 2011 (21926110)	Mixed ancestry South African	201	0.139	427	0.183		NS
*FASL*	rs763110 (-844 T > C)	Bye et al., 2011 (21926110)	Black South African	358	0.192	477	0.189		NS
		Bye et al., 2011 (21926110)	Mixed ancestry South African	201	0.416	427	0.386		NS
*GSTP1*	rs1695 (Ile105Val)	Matejcic et al., 2011	Black South African	325	0.518	474	0.534		NS
	rs1695 (Ile105Val)	Matejcic et al., 2011	Mixed ancestry South African	229	0.454	428	0.438		NS
	rs1695 (Ile105Val)	Li et al., 2010 (20540773)	Black South African		0.39		0.37		NS
	rs1695 (Ile105Val)	Li et al., 2010 (20540773)	Mixed ancestry South African		0.38		0.41		NS
	rs1138272 (Ala114Val)	Li et al., 2010 (20540773)	Black South African		0.22		0.07		NS
	rs1138272 (Ala114Val)	Li et al., 2010 (20540773)	Mixed ancestry South African		0.19		0.03		NS
*GSTT1*	Deletion allele	Matejcic et al., 2011 (22216261)	Black South African	311	0.574	462	0.554		NS
		Matejcic et al., 2011 (22216261)	Mixed ancestry South African	217	0.493	414	0.495		NS
*GSTT2B*	Deletion allele	Matejcic et al., 2011 (22216261)	Black South African	320	0.336	461	0.371		NS
		Matejcic et al., 2011 (22216261)	Mixed ancestry South African	226	0.418	425	0.501		OR = 0.71 (0.57–0.90); p = 0.004
*MGMT*	rs12917 (Leu84Phe)	Bye et al., 2011 (21926110)	Black South African	358	0.189	477	0.195		NS
		Bye et al., 2011 (21926110)	Mixed ancestry South African	201	0.222	427	0.168		OR = 1.41 (1.05–1.91); p = 0.023
*MLH1*	rs13320360 (c.546-191T/C)	Vogelsang et al., 2012 (22623965)	Black South African	343	0.15	340	0.17		NS
		Vogelsang et al., 2012 (22623965)	Mixed ancestry South African	203	0.07	264	0.06		NS
*MLH3*	rs28756991 (Arg797His)	Vogelsang et al., 2012 (22623965)	Black South African	345	0.11	342	0.12		NS
		Vogelsang et al., 2012 (22623965)	Mixed ancestry South African	205	0.09	264	0.4	G	OR = 2.07 (1.04–4.12); p = 0.038
*MSH2*	rs17217772 (Asn127Ser)	Vogelsang et al., 2012 (22623965)	Black South African	341	0.06	343	0.06		NS
		Vogelsang et al., 2012 (22623965)	Mixed ancestry South African	204	0.03	264	0.03		NS
	rs10188090 (c.2635-765G/A)	Vogelsang et al., 2012 (22623965)	Black South African	343	0.09	342	0.10		NS
		Vogelsang et al., 2012 (22623965)	Mixed ancestry South African	205	0.31	265	0.33		NS
	rs3771280 (c.1510+118T/C)	Vogelsang et al., 2012 (22623965)	Black South African	344	0.11	339	0.12		NS
		Vogelsang et al., 2012 (22623965)	Mixed ancestry South African	202	0.35	266	0.37		NS
*MSH3*	rs26279 (Ala1045Thr)	Vogelsang et al., 2012 (22623965)	Black South African	341	0.40	344	0.43		NS
		Vogelsang et al., 2012 (22623965)	Mixed ancestry South African	204	0.38	263	0.32	A	OR = 2.71 (1.34–5.50); p = 5.71×10^-3^
	rs1428030 (c.1341-12568A/G)	Vogelsang et al., 2012 (22623965)	Black South African	342	0.29	342	0.27		NS
		Vogelsang et al., 2012 (22623965)	Mixed ancestry South African	201	0.23	264	0.20		NS
	rs1805355 (Pro231Pro)	Vogelsang et al., 2012 (22623965)	Black South African	343	0.28	339	0.29		NS
		Vogelsang et al., 2012 (22623965)	Mixed ancestry South African	203	0.24	265	0.22		NS
*NAT1*	rs1057126 (1088T > A NAT1*10)	Matejcic et al., 2015 (26447020)	Black South African	463	54.8	480	57.7		NS
		Matejcic et al., 2015 (26447020)	Mixed ancestry South African	269	43.4	288	40.1		NS
	rs15561 (1095C > A NAT1*10, NAT1*3)	Matejcic et al., 2015 (26447020)	Black South African	463	55.7	480	57.7		NS
		Matejcic et al., 2015 (26447020)	Mixed ancestry South African	269	46.5	288	43		NS
*NAT2*	rs1799930 (590G/A NAT2*6)	Matejcic et al., 2015 (26447020)	Black South African	463	24.7	480	21.4		NS
		Matejcic et al., 2015 (26447020)	Mixed ancestry South African	269	22.4	288	22		NS
	rs1801280 (341T/C NAT2*5)	Matejcic et al., 2015 (26447020)	Black South African	463	27.1	480	29		NS
		Matejcic et al., 2015 (26447020)	Mixed ancestry South African	269	25.2	288	33.2	C	0R = 0.57 (0.38–0.87) p = 0.01
	rs1799931 (857G/A NAT2*7)	Matejcic et al., 2015 (26447020)	Black South African	463	0.01	480	0.05		Not informative
		Matejcic et al., 2015 (26447020)	Mixed ancestry South African	269	0.05	288	0.04		NS
	rs1801279 (191G/A NAT2*14)	Matejcic et al., 2015 (26447020)	Black South African	463	0.053	480	0.063		NS
		Matejcic et al., 2015 (26447020)	Mixed ancestry South African	269	0.038	288	0.023		NS
*UNC5CL*	rs10484761 (G/A)	Bye et al., 2012 (22865593)	Black South African	407	0.467	849	0.477		NS
		Bye et al., 2012 (22865593)	Mixed ancestry South African	257	0.354	860	0.314		NS
*PTGS2*	rs20417 (-765 G/C)	Bye et al., 2011 (21926110)	Black South African	358	0.471	477	0.513		NS
		Bye et al., 2011 (21926110)	Mixed ancestry South African	201	0.376	427	0.321		NS
	rs689466 (-1195 A/G)	Bye et al., 2011 (21926110)	Black South African	358	0.064	477	0.053		NS
		Bye et al., 2011 (21926110)	Mixed ancestry South African	201	0.103	427	0.155	G	OR = 0.63 (0.43–0.91); p = 0.014
*PDE4D*	rs10052657 (C/A)	Bye et al., 2012 (22865593)	Black South African	407	0.137	849	0.128		NS
		Bye et al., 2012 (22865593)	Mixed ancestry South African	257	0.175	860	0.155		NS
*PLCE1*	rs2274223 (His1927Arg)	Bye et al., 2012 (22865593)	Black South African	407	0.416	849	0.403		NS
		Bye et al., 2012 (22865593)	Mixed ancestry South African	257	0.437	860	0.40		NS
	rs17417407 (Arg548Leu)	Bye et al., 2012 (22865593)	Black South African	407	0.166	849	0.211	T	OR = 0.74 (0.60–0.93); p = 0.008
		Bye et al., 2012 (22865593)	Mixed ancestry South African	257	0.174	860	0.18		NS
	rs1438095332 (5’UTR 14 bp indel)	Bye et al., 2012 (22865593)	Black South African	321	0.234	456	0.242		NS
	rs199781223 (Gly1199Ser)	Bye et al., 2012 (22865593)	Black South African	321	0.053	449	0.045		NS
	rs3765525^3^ (Ile1777Thr)	Bye et al., 2012 (22865593)	Black South African	316	0.472	452	0.463		NS
	rs58539480 (Pro1890Leu)	Bye et al., 2012 (22865593)	Black South African	307	0.073	429	0.064		NS
	rs17417407 (G/T)	Chen et al., 2019 (30753320)	Black South African^4^	591	0.17	852	0.21	T	OR = 0.76 (0.60–0.95); p = 0.014
		Chen et al., 2019 (30753320)	Black South African^5^	880	0.19	939	0.19		NS
	rs7084339 (G/A)	Chen et al., 2019 (30753320)	Black South African^5^	880	0.48	939	0.46		NS
	rs3765524 (T/C)	Chen et al., 2019 (30753320)	Black South African^4^	591	0.47	852	0.47		NS
		Chen et al., 2019 (30753320)	Black South African^5^	880	0.48	939	0.46		NS
	rs2274223 (A/G)	Chen et al., 2019 (30753320)	Black South African^4^	591	0.42	852	0.40		NS
		Chen et al., 2019 (30753320)	Black South African^5^	880	0.41	939	0.43		NS
	rs11187850 (A/G)	Chen et al., 2019 (30753320)	Black South African^5^	880	0.21	939	0.19		NS
*PMS1*	rs5742938 (c.-21+639G/A)	Vogelsang et al., 2012 (22623965)	Black South African	345	0.18	344	0.15		NS
		Vogelsang et al., 2012 (22623965)	Mixed ancestry South African	203	0.43	266	0.48	A	OR = 1.73 (1.07–2.79); p = 0.027
	rs13404927 (c.699+3331G/A)	Vogelsang et al., 2012 (22623965)	Black South African	342	0.18	339	0.19		NS
		Vogelsang et al., 2012 (22623965)	Mixed ancestry South African	204	0.14	264	0.12		NS
*RUNX1*	rs2014300 (A/G)	Bye et al., 2012 (22865593)	Black South African	407	0.378	849	0.403		NS
		Bye et al., 2012 (22865593)	Mixed ancestry South African	257	0.438	860	0.370	G	OR = 1.33 (1.09–1.63); p = 0.0055
	rs2014300 (A/G)	Chen et al., 2019 (30753320)	Black South African^4^	591	0.38	852	0.40		NS
		Chen et al., 2019 (30753320)	Black South African^5^	880	0.36	939	0.36		NS
	rs2834718 (T/A)	Chen et al., 2019 (30753320)	Black South African^5^	880	0.33	939	0.33		NS
*SLC11A1*	-237C/T	Zaahl et al., 2005 (15860357)	Mixed ancestry South African	105	0.029	110	0.1		p < 0.004
	-8G/A	Zaahl et al., 2005 (15860357)	Mixed ancestry South African	105	0.004	110	0.009		NS
	IVSI-28C/T	Zaahl et al., 2005 (15860357)	Mixed ancestry South African	105	0.028	110	0.0004		p < 0.05
	GT-repeat	Zaahl et al., 2005 (15860357)	Mixed ancestry South African		0.171		0.191		NS
*SULT1A1*	638G/A in Exon 7	Dandara et al., 2006 (16272171)	Black South African	145	0.42	194	0.37		NS^1^
		Dandara et al., 2006 (16272171)	Mixed ancestry South African	100	0.40	94	0.29		NS
*TMEM173*	rs13181561 (A/G)	Chen et al., 2019 (30753320)	Black South African^5^	880	0.48	939	0.49		NS
	rs13153461 (G/A)	Chen et al., 2019 (30753320)	Black South African^4^	591	0.04	852	0.05		NS
*TP53*	16-bp insertion in intron 3	Vos et al., 2003 (12550754)	Black South African	74	0.108	118	0.364		
	rs200073907 (Exon 4 codon 34)	Vos et al., 2003 (12550754)	Black South African	74	0.115	118	0.102		NS
	rs750578863 (Exon 4 codon 36)	Vos et al., 2003 (12550754)	Black South African	73	0.089	115	0.143		NS
	Arg72Pro	Vos et al., 2003 (12550754)	Black South African	73	0.356	115	0.409		p < 0.05
	Arg72Pro	Eltahir et al., 2012 (23053979)	Sudanese	25	0.49	235	0.51		NS
	rs1800371 (G/A)	Chen et al., 2019 (30753320)	Black South African^4^	591	0.02	852	0.03		NS
		Chen et al., 2019 (30753320)	Black South African^5^	880	0.03	939	0.02		NS
*XBP1*	rs2239815 (C/T)	Chen et al., 2019 (30753320)	Black South African^4^	591	0.21	852	0.16	T	OR = 1.41 (1.15–1.74) p = 0.001
		Chen et al., 2019 (30753320)	Black South African^5^	880		939			NS
*^1^* Increased risk among smokers with SULT1A1*2/*2 genotype, but sample size was small. *^2^* *When OR > 1, effect allele = increased risk; when OR < 1, effect allele = protective effect.* *^3^* *rs3765525 has been merged into rs959421.* *^4^* *Western and Eastern Cape Province Black Population.* *^5^* *Gauteng Province Black Population.*									

**Table 5** shows a summary of the pathways for the 20 genes. All the genes encode for proteins. Three of the genes, *ADH1B, ADH3,* and *ALDH2*, are involved in alcohol metabolism (Li et al., 2008; Bye et al., 2011). Three mismatch repair genes, *MLH3, MSH3,* and* PMS1*, play a role in genomic integrity (Vogelsang et al., 2012). They are reported to also play a role in carcinogenesis. MGMT is involved in cell defense against mutagens, and mutations in the gene are reported to be associated with cancer formation (Bye et al., 2011). *NAT2* and *GSTT2B* play a role in the activation and deactivation of drugs and carcinogens, with reports of mutations being associated with carcinogenesis (Matejcic et al., 2015). Genes regulating cell apoptosis are *TP5, CHEK2, and CASP8* (Vos et al., 2003; Bye et al., 2011; Eltahir et al., 2012; Chen et al., 2019). *TP53* and *CHEK2* are also involved in gene expression and DNA repair. Regulation of gene expression is facilitated by *PLCE1* and *SLC11A1* (Zaahl et al., 2005; Bye et al., 2012). The *AR* gene regulates the sex hormones, androgens (Dietzsch et al., 2003), while *CYP2E1* and *CYP3A5* are involved in steroid, cholesterol, and lipid synthesis (Dandara et al., 2005; Li et al., 2005; Chelule et al., 2006). *CYP2E1* also metabolizes drugs and has been implicated in carcinogenesis. *CP* facilitates transportation of iron from organs into the blood cells; *RUNX1* plays a role in hematopoiesis and *PTGS2* in inflammation and mitogenesis (Bye et al., 2011; Bye et al., 2012; Strickland et al., 2012).

**Table 5:** Biological pathways for genetic susceptibility studies showing putative association with ESCC in African populations.

Gene	Full name	Pathway
*ADH1B*	Alcohol dehydrogenase 1B (class I), beta polypeptide	Ethanol metabolism
*ADH3*	Alcohol dehydrogenase ADH3	Metabolizes ethanol into acetaldehyde
*ALDH2*	Aldehyde dehydrogenase 2 family member	Alcohol metabolism. Implicated in increased susceptibility for cancer
*AR*	Androgen receptor	Regulates binding of androgens on androgen receptor
*CASP8*	Caspase 8	Cell apoptosis
*CHEK2*	Checkpoint kinase 2	Tumor suppressor gene. Mutations associated with predisposition to carcinogenesis
*CP*	Ceruloplasmin	Peroxidation of iron through its transportation from organs and tissue into blood
*CYP2E1*	Cytochrome P450 family 2 subfamily E member 1	Drug metabolism and catalysis and synthesis of cholesterol, steroids, and other lipids. Implicated in cancer development
*CYP3A5*	cytochrome P450 family 3 subfamily A member 5	Involved in drug metabolism and in the synthesis of cholesterol, steroids, and other lipids
*GSTT2B*	Glutathione S-transferase theta 2B (gene/pseudogene)	Conjugation of glutathione to electrophilic and hydrophobic compounds. Plays a role in carcinogenesis
*MGMT*	O-6-methylguanine-DNA methyltransferase	DNA repair and defense from alkylating agents which cause mutagenesis and toxicity. Implicated in several cancers.
*MLH3*	MutL homolog 3	Maintenance of genomic integrity following cell division and DNA replication. Germline mutations implicated in cancer and somatic mutations implicated in microsatellite instability
*MSH3*	MutS homolog 3	Forms heterodimers with MSH2. Involved in mismatch repair and implicated in cancer development.
*NAT2*	N-acetyltransferase 2	Activation and deactivation of arylamine and hydrazine drugs and carcinogens. Implicated in high cancer incidence and drug toxicity.
*PTGS2*	Prostaglandin-endoperoxide synthase 2	A dioxygenase and a peroxidase involved in both inflammation and mitogenesis
*PLCE1*	Phospholipase C epsilon 1	Regulation of cell growth, differentiation, and gene expression.
*PMS1*	PMS1 homolog 1, mismatch repair system component	Mismatch repair gene. Mutations implicated in cancer development.
*RUNX1*	Runt related transcription factor 1	Development of hematopoiesis
*SLC11A1*	Solute carrier family 11 (proton-coupled divalent metal ion transporter), member 1	Regulation of gene expression.
*TMEM173*	Transmembrane protein 173	Regulation of the innate immune response to viral and bacterial infections. Role in tumorigenesis still inadequate
*TP53*	Tumor protein 53	Regulation of gene expression, cell cycle, apoptosis, and DNA repair.
*XBP1*	X-box binding protein 1	Regulation of genes involved in endoplasmic reticulum protein synthesis, folding, glycosylation, redox metabolism, autophagy, lipid biogenesis, and vesicular trafficking. Associated with development of cancer.

Nine of the 25 associated SNPs were from small studies with fewer than 150 cases and controls. These SNPs are in the following six genes: *ADH3, AR, CP, CYP3A5, SLC11A1*, and *TP53*. Because of the small sample size, the reliability and replicability of these results are uncertain. Sixteen of the SNPs came from studies with at least 150 cases and controls, and one study with 142 cases. These sample sizes could potentially give reliable and replicable results. The 16 SNPs were from the following genes: *ADH1B, ALDH2, CASP8, CHEK2, CYP2E1, GSTT2B, MGMT, MLH3, MSH3, NAT2, PLCE1, PMS1, PTGS2, and RUNX1*.

Two of the 16 SNPs are in the *ALDH2* gene**and were analyzed in two different studies. However, it is not clear whether these two SNPs are the same because, while one study reported the NCBI rs number (rs886205) (Bye et al., 2011), the other study did not (Li et al., 2008).The two SNPs reported very different MAF, and opposite odds ratios of 2.35 and 0.70 demonstrating increased risk and a protective effect, respectively.

**Figure 2:**
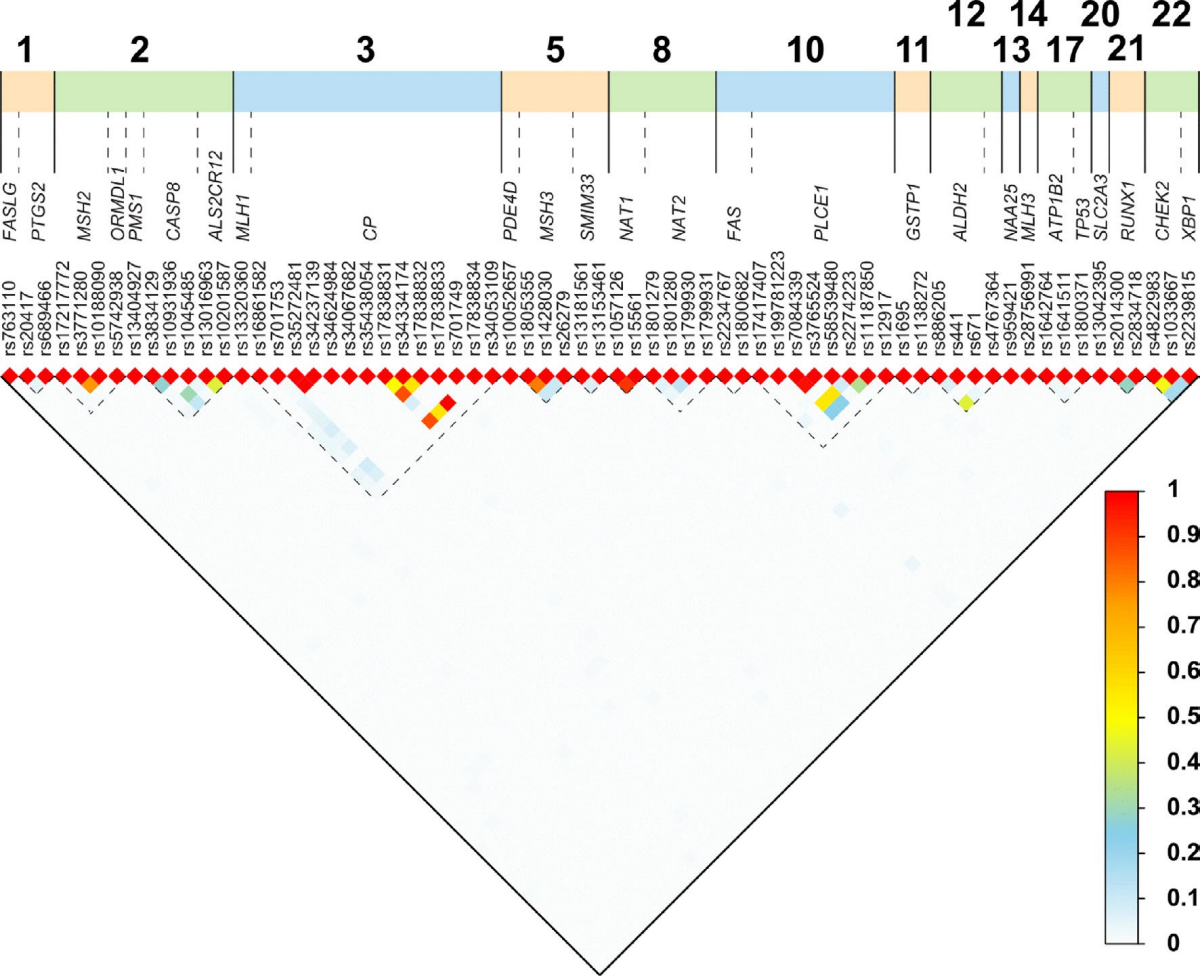
Linkage disequilibrium (LD) plot for paired SNPs. We obtained the rs numbers of the variants from dbSNP (version 152; April 2019; (https://www.ncbi.nlm.nih.gov/snp/)) and used the canonical SNP identifier. To determine the LD between the SNPs, we obtained the imputed data set from the Thousand Genomes project (1000 Genomes Release Phase 3 2013-05-02) and used bcftools to extract all individuals from African populations not including African Americans, and the 77 SNPs discussed here using all synonyms (alternative rs IDs) for SNPs (Auton et al., 2015). We obtained a dataset of 504 individuals and 67 SNPs. We computed all pair-wise r2 using PLINK (v1.09) (Danecek et al., 2011; Chang et al., 2015).

Six of the 16 SNPs were reported to reduce the risk of ESCC, and they are the following: *ADH1B* (Arg48His; rs1229984), *ALDH2* (+82 A > G; rs886205), *GSTT2B* (deletion allele), *NAT2* (341T > C; rs1801280), *PTGS2* (-1195 A > G; rs689466), and *PLCE1* (Arg548Leu; rs17417407). The remaining 10 SNPs were reported to increase the risk of ESCC: *ALDH2* (ALDH2*1/*2), *CASP8* (Asp302His; rs1045485), *CHEK2* (rs4822983 C > T, and rs1033667, C > T), *CYP2E1* (7632T > A), *MGMT* (Leu84Phe; rs12917), *MLH3* (Arg797His; rs28756991), *MSH3* (Ala1045Thr; rs26279), *PMS1* (c.-21+639G > A; rs5742938), and *RUNX1* (rs2014300). Eleven of the 16 SNPs showed association in the South African Admixed population, while only four showed association in the Black South African population and one in a combined South African population. All the studies used PCR-based methods for genotyping. Using the 1000 Genomes Database, r^2^ analysis was carried out on SNPs reported in the same gene, to assess the LD between the SNPs. Thirteen pairs of SNPs in *MHS2, CP, MSH3, PLCE1,CHEK2,* and *NAT1* genes had r^2^ > 0.45, shown in **Figure 2** and **Table S3**.

Altogether 44 somatic changes were reported in the following 22 genes: *AR*, *CCND1*, *CDKN2A*, *COL1A2*, *EFGR*, *EP300*, *FAT1*, *FAT2*, *FAT3*, *FAT4*, *FBXW7*, *JAG1*, *KMT2C* *(MLL3)*, *KMT2D* *(MLL2)*, *MUC2*, *NFE2L2*, *NOTCH1*, *NOTCH3*, *PIK3CA*, *SERPINB4*, *TP53*, and *TP63*, and six genetic loci without specific gene names (**Table 6**). The specific locus positions with the corresponding microsatellite markers are as follows: 2p (D2S123), 3p13 (D3S659), 3p24.2-25 (D3S1255), 4q12 (Bat 25), 2p21-p16.3 (Bat 26), and 1p12-13.3 (Bat 40). These variants were reported in the South African (20 variants), Kenyan (three variants), and Malawian (21 variants) populations. While the majority of the studies used PCR-based methods, a more recent study used WES as the analysis method (Liu et al., 2016). A total of 18 of the 22 genes with somatic variants in cancer tissue were discovered using WES. Statistical significance was not reported for any of the 44 variants. The most common type of somatic variants was missense mutations, reported in 14 of the 22 genes (64%) (Patel et al., 2011; Liu et al., 2016). Other somatic changes included copy number gains (14%), copy number losses (5%), deletions (14%), insertions (14%), and frameshift mutations (14%). In three studies (Dietzsch and Parker, 2002; Dietzsch et al., 2003; Naidoo et al., 2005), microsatellite instability and loss of heterozygosity (LOH) were reported (14%).

**Table 6:** Summary of studies investigating somatic changes linked to ESCC in African patients.

Gene	Study (PMID)	Population	Findings
*AR*	Dietzsch et al., 2003 (12925954)	Black and mixed ancestry South African	LOH at CAG locus
*CCND1*	Liu et al., 2016 (29148985)	Malawian	Enriched copy number gains
*CDKN2A*	Gamieldien et al., 1998 (9808520)	Black South African	Insertions Deletions Frameshift mutations
	Liu et al., 2016 (29148985)	Malawian	Copy number losses
*COL1A2*	Dietzsch and Parker, 2002 (12435113)	Black South African	LOH (promoter and 1^st^ intron) No evidence of MSI or allelic amplification
*EFGR*	Liu et al., 2016 (29148985)	Malawian	Copy number gains
*EP300*	Liu et al., 2016 (29148985)	Malawian	Missense mutations
*FAT1*	Liu et al., 2016 (29148985)	Malawian	Nonsense mutations
*FAT2*	Liu et al., 2016 (29148985)	Malawian	Missense mutations
*FAT3*	Liu et al., 2016 (29148985)	Malawian	Missense mutations
*FAT4*	Liu et al., 2016 (29148985)	Malawian	Missense mutations
*FBXW7*	Liu et al., 2016 (29148985)	Malawian	Frameshift mutations
*JAG1*	Liu et al., 2016 (29148985)	Malawian	Missense mutations
*KMT2C (MLL3)*	Liu et al., 2016 (29148985)	Malawian	Missense mutations
*KMT2D (MLL2)*	Liu et al., 2016 (29148985)	Malawian	Nonsense mutations
Mismatch repair genes	Naidoo et al., 2005 (15735161)	South African	LOH and MSI at: D2S123 (2p) D3S659 (3p13) D3S1255 (3p3p24.2-25) Bat 25 (4q12) Bat 26 (2p2p21-p16.3) Bat 40 (1p12-13.3)
*MUC2*	Liu et al., 2016 (29148985)	Malawian	Missense mutations
*NFE2L2*	Liu et al., 2016 (29148985)	Malawian	Missense mutations
*NOTCH1*	Liu et al., 2016 (29148985)	Malawian	Missense mutations
*NOTCH3*	Liu et al., 2016 (29148985)	Malawian	Missense mutations
*PIK3CA*	Liu et al., 2016 (29148985)	Malawian	Missense mutations
*Ras genes*	Victor et al., 1990 (2199031)	South African	No mutations found in codon 12, 13 or 61
*SERPINB4*	Liu et al., 2016 (29148985)	Malawian	Missense mutations
*TP53*	Liu et al., 2016 (29148985)	Malawian	Missense and nonsense mutations
	Gamieldien et al., 1998 (9808520)	Black South African	Exon 5–8 frameshift mutations: point mutations, deletions and insertions
	Patel et al., 2011 (22040862)	Kenyan	Exon 5–8 mutations: missense, nonsense and deletions
	Vos et al., 2003 (12550754)	South African	16-bp insertion in intron 3
	Vos et al., 2003 (12550754)	South African	Exon 4 polymorphism in codons 34, 36 and 72 LOH (16-bp repeat locus)
*TP63*	Liu et al., 2016 (29148985)	Malawian	Copy number gains

LOH, loss of heterozygosity; MSI, microsatellite instability.

**Table 7** shows a summary of the pathways in the 22 genes reporting somatic changes. Five genes, *AR, EP300, KMT2D, KMT2C*, and *TP53*, play a role in the regulation of transcription (Gamieldien et al., 1998; Dietzsch et al., 2003; Vos et al., 2003; Patel et al., 2011; Liu et al., 2016). The encoded protein for the *AR* gene functions as a steroid hormone activated transcription factor, while KMT2D has a role in methylation. Both *TP53* and *EP300* have been implicated in a number of cancers (Gamieldien et al., 1998; Vos et al., 2003; Patel et al., 2011; Liu et al., 2016). *TP53* additionally functions in DNA repair, gene expression, and apoptosis. The mismatch repair genes also facilitate DNA repair (Naidoo et al., 2005). *CCND1, CDKN2A, FAT1/2/3/4*, and *Ras* genes are all reported to be involved in cell cycle pathways including regulation of mitotic events, cell proliferation, and cell growth and death (Victor et al., 1990; Gamieldien et al., 1998; Liu et al., 2016). *NOTCH1* and *NOTCH3* both facilitate cell and tissue development (Liu et al., 2016). *JAG1* plays a role in hematopoiesis while *NFE2L2* is involved in response to inflammation including production of free radicals (Liu et al., 2016). *PIK3CA* is an oncogene implicated in tumor development while *SERPINB4* modulates response against tumor cells (Liu et al., 2016). *EGFR* and *COL1A2* genes encode for epidermal growth factor and type 1 collagen, respectively (Dietzsch and Parker, 2002; Liu et al., 2016). *FBXW7* is a tumor suppressor involved in ubiquitin degradation (Liu et al., 2016). *MUC2* facilitates the formation of a mucous barrier that protects the gut lumen (Liu et al., 2016). *TP63* gene is involved in tissue and organ development including skin and heart, and in adult stem cell regulation (Liu et al., 2016).

**Table 7:** Biological pathways for somatic changes studies showing putative association with ESCC in African populations.

Gene	Full name	Pathway
*AR*	Androgen receptor gene	Regulation of gene expression and the protein functions as a steroid-hormone activated transcription factor.
*CCND1*	Cyclin D1	Regulators of CDK kinases and mitotic events. Mutations and overexpression of the gene has been associated with cancer development.
*CDKN2A*	Cyclin dependent kinase inhibitor 2A	A tumor suppressor gene which regulates the cell cycle. Commonly inactivated in a variety of tumors.
*CHEK2*		
*COL1A2*	Collagen type I, alpha 2 chain	Encodes for type I collagen, which is an abundant connective tissue protein and part of extracellular matrix.
*EFGR*	Epidermal growth factor receptor	Encodes for the growth factor epidermal growth factor receptor.
*EP300*	E1A binding protein p300	Encodes the adenovirus E1A-associated cellular p300 transcriptional co-activator protein which functions in transcription regulation. Mutations have been implicated in tumorigenesis.
*FAT1/2/3/4*	FAT atypical cadherin 1/2/3/4	Human homologues of the *Drosophila* FAT genes. Putative tumor suppressor involved in cell proliferation during *Drosophila* development.
*FBXW7*	F-box and WD repeat domain containing 7	Encodes an F-Box protein which binds directly to cyclin E and potentially targets cyclin E for ubiquitin-mediated degradation.
*JAG1*	Jagged 1	Encodes for the human homolog of the *Drosophila* jagged 1 protein which is involved in hematopoiesis.
*KMT2C (MLL3)*	Lysine methyltransferase 2C	The gene is member of the myeloid/lymphoid or mixed-lineage leukemia (MLL) family. It encodes a nuclear protein involved in transcriptional regulation.
*KMT2D (MLL2)*	Lysine methyltransferase 2D	Methylation of histones and transcriptional regulation.
Mismatch repair genes	Mismatch repair genes	DNA repair. Mutations have been implicated in cancer.
*MUC2*	Mucin 2, oligomeric mucus/gel-forming	Formation of insoluble mucous barrier that protects the gut lumen.
*NFE2L2*	Nuclear factor, erythroid 2 like 2	Encodes for proteins involved in response to inflammation including free radical production.
*NOTCH1*	NOTCH1	Development of cell and tissue. Mutations have been reported to be linked with tumorigenesis.
*NOTCH3*	NOTCH3	The third discovered human homologue of the *Drosophila* melanogaster type I membrane protein notch. Involved in intercellular signaling pathways in neural development.
*PIK3CA*	Phosphatidylinositol-4,5-bisphosphate 3-kinase catalytic subunit alpha	Oncogenic and implicated in cancer development.
*Ras genes*	Rat sarcoma	Regulation of cell signaling pathways, and cell growth and death.
*SERPINB4*	Serpin family B member 4	Inactivation of granzyme M, an enzyme that kills tumor cells. Highly expressed in tumor cells.
*TP53*	Tumor protein p53	Regulates transcription, expression of target genes, thereby inducing cell cycle arrest, apoptosis, senescence, DNA repair, or changes in metabolism. Implicated in a number of cancers.
*TP63*	Tumor protein p63	Involved in the following processes in skin development and maintenance, adult stem/progenitor cell regulation, heart development, and premature aging.

### Interaction Studies

Combinations of specific genotypes with environmental factors were also reported to be associated with ESCC in a number of studies (**Table 2**). The main two environmental factors studied were smoking and alcohol consumption. The interaction between smoking and alcohol status and specific genotypes was measured and reported as frequency (percentage) and assessed using p values and odds ratios in nine genetic susceptibility studies (Dandara et al., 2005; Li et al., 2005; Li et al., 2010; Dandara et al., 2006; Li et al., 2008; Li et al., 2010; Bye et al., 2011; Matejcic et al., 2011; Vogelsang et al., 2012; Matejcic et al., 2015). Four studies showed statistically significant associations between both alcohol and smoking status and variants in the *CYP3A5, CYP2E1, GST*, and *NAT2* genes (Dandara et al., 2005; Li et al., 2005; Matejcic et al., 2015). *SULT1A1* variants were associated with smoking status only (Dandara et al., 2006). Other interaction studies included wood/charcoal use and mutations in the *GST* genes (Li et al., 2010), as well as red and white meat intake and SNPs in *NAT1/2* genes (Matejcic et al., 2015).

## Discussion

### General Systematic Review Findings

In this study, we systematically evaluated the genetic variants reported to be associated with ESCC in African populations providing the first systematic review on genetic factors of ESCC in this region. Of all studies that have been published on genetic association to ESCC in the African populations, only 23 fit our selection criteria. It was clear from the beginning that there is a dearth of information on this topic. Our analysis showed that 25 germline SNPs were reported to be associated with ESCC in the South African population. However, none of these SNPs were repeated in three or more independent studies; hence, a meta-analysis was not possible. Additionally, only three (*ALDH2, PLCE* and *CYP2E1*) of the 20 genes were analyzed in two independent studies, but testing for different SNPs. We determined that it was unlikely that the two *ALDH2* SNPs analyzed were the same SNPs. This is because the MAFs were significantly different and, while one SNP had a protective effect (reduced risk), the other increased risk. The lack of studies re-assessing the same genetic variants poses a major hurdle in validating existing evidence on the association between genetic variants and ESCC development. This makes resolving the genetic etiology of ESCC in African populations difficult.

### Genetic Susceptibility to ESCC

Of the 25 SNPs from the genetic susceptibility studies that showed an association to ESCC, we concluded that results on 16 SNPs had the potential to be reliable and reproducible due to the larger sample sizes. Ten of the SNPs were reported to increase the risk of ESCC, while six were reported to reduce the risk. However, it was noted that the majority (11) of these SNPs showed association in the South African Admixed population and the studies did not report controlling for population stratification. This is a highly admixed population (Chimusa et al., 2013), in which the predominant ancestral lines are Khoesan (32–43%), Bantu-speaking Africans (20–36%), European (21–28%), and Asian (9–11%) (De Wit et al., 2010). This diverse population is a result of South Africa’s colonial and trade history, and constitutes 9% of the total South African population (De Wit et al., 2010). Genetic variability can also be seen in the Black South African population (Chimusa et al., 2013). Without controlling for population stratification, the reproducibility of these results is questionable. It is, however, important to note that the majority of these studies were carried out several years ago, and information on population stratification and methods to detect it may not have been available as yet.

Re-examination of common SNPs from the Chinese population was done in three of the studies (Bye et al., 2011; Bye et al., 2012; Chen et al., 2019), but the findings were not conclusive. It is possible that there may be population-specific differences influencing the genetic etiology of ESCC in the African populations. This may also point to the role of environmental factors contributing to the genetic susceptibility to ESCC through gene-environment interactions.

### Somatic Changes in ESCC

Forty-four somatic variants were reported, but only two were significantly associated with ESCC. The paucity of information was also evident in the somatic variant studies. There were significantly fewer studies (8) on somatic variants than on genetic susceptibility (17). The molecular profiling of tumors is of great importance as it is relevant in the development of targeted cellular therapeutics. One gene (*CDKN2A*) was analyzed in two studies, but these studies focused on a different variant. Another gene, TP53, was analyzed in four studies, but two studies analyzed different parts of the gene, and two had no control data. It was evident, however, that the WES study provided with a wider variety of genetic variants associated with ESCC (Liu et al., 2016). The WES study overall had the largest number of genetic variants of all the 23 studies and was able to identify variants in an unbiased manner.

### Common Limitations Among the African Studies

There were no GWAS among the studies we analyzed, but reports from the Chinese and European studies demonstrated that GWAS are able to successfully identify common genetic variants associated with ESCC (Abnet et al., 2017). To date, GWAS has successfully identified more than 700 loci for cancer risk. However, these studies have been predominantly done in populations of European ancestry (80%), with African and Latin American populations contributing less than 1% (Van Loon et al., 2018). A shift to WES and GWAS on the African populations might, therefore, yield better results in identifying variants that play a role in ESCC development. The African Esophageal Cancer Consortium, which was initiated in 2016 by African investigators and International partners, released a call to action to, among other priority activities, increase molecular research on esophageal cancer in Africa, particularly GWAS and genomic profiling (Van Loon et al., 2018).

One of the main deficiencies in the studies was that the majority of the genetic susceptibility studies did not report a power calculation, or a genotyping error, and this may have resulted in studies being underpowered and with increased type II error. Few studies reported correction for multiple testing; however, many of the studies were not analyzing multiple variants at the same time. The lack of correction for multiple testing, therefore, is not a reflection on the methodological quality. Very few studies reported NCBI rs numbers. In most studies, the diagnosis of ESCC in patients was adequately defined with no ambiguity on the number of patients with ESCC. There were, however, three studies that combined samples from patients with squamous cell and adenocarcinoma into one case group, which could introduce bias (Dietzsch et al., 2003; Eltahir et al., 2012; Vogelsang et al., 2012).

It is important to note that rs numbers were poorly documented in the majority of the studies assessed in this systematic review. Additionally, in many of these studies, the positions of the SNPs using genome coordinates were not reported, hence making it difficult to locate the SNPs. In the absence of an rs number, we recommend that authors report the position using genome coordinates and the version of the genome used as a reference.

The somatic variant studies also had adequately defined ESCC diagnosis for the majority of the studies. While the variant classification and type were reported by most studies, there was no confirmation of the results (except for two studies). Overall, for both the germline and somatic variant studies, the quality of reporting for the majority of the studies was not adequate. Other important limitations and biases are the lack of controlling for population stratification and small sample sizes in the study populations, which may have led to unreliable results.

### Limitations of the Systematic Review

While we did a comprehensive search in four of the main literature databases, it is possible that we could have missed some non-English studies on African populations. Because of the lack of replication and validation studies, we could not carry out a meta-analysis in the current study. Furthermore, we did not re-analyze the data and relied on reported p values and odds ratios for descriptive analysis.

## Conclusions

While this review has highlighted a number of genes that may be potentially associated with ESCC in the African populations, limitations such as lack of reproducibility, quality of reporting, and quality of assessment remain a major concern. The implications of having these inconsistencies and lack of reproducibility are that the genetic etiology of ESCC in Africa will continue to be unclear. The region lags behind in contributing to genetic knowledge and literature on ESCC. Importantly, any preventative, diagnostic, or therapeutic interventions cannot be effectively identified or applied in these populations.

The identification of genetic markers of esophageal cancer susceptibility has clear translational benefits to African populations in understanding the underlying disease risk and heritability. Benefits include the utilization of genetic information to improve risk prediction, which can be translated into prevention and screening programs relevant and specific to the African population. These studies also play a role in identifying and quantifying the interactions of modifiable environmental risk factors, which interact with these genetic variants, and hence provide a platform for better targeted interventions. The ability to sufficiently translate genetic research on the African population is dependent on more genetic studies done on the population.

Our recommendations are that more and larger genetic studies be done on the African populations, particularly focusing on WES and GWAS approaches. This will require multinational collaborations between the African countries.

## Ethics Statement

The study was approved by the Stellenbosch University Health Research Ethics Committee as part of the Doctoral Studies of HS (HREC Reference #: S18/10/250).

## Author Contributions

VL, VS, and HS carried out literature searches. HS, VS, and HK appraised the articles, summarized the results, prepared the tables and figures, and drafted the manuscript. VS and VL reviewed the articles and edited the manuscript. VS and HK conceptualized the idea for the research, obtained funding, supervised the project, and wrote sections of the manuscript. VL provided specialist expertise and knowledge, and critically reviewed the manuscript. GT carried out the r^2^ analyses, prepared the r^2^ figure and table, and critically reviewed and revised the manuscript. All authors approved the final version of the manuscript.

## Funding

This work was supported by the African Cancer Institute, Faculty of Medicine and Health Sciences, Stellenbosch University. HS acknowledges the Beit Trust Hardship Fund for providing a Doctoral Scholarship in part aid of tuition and registration fees and the Collaboration for Evidence-based Healthcare and Public Health in Africa (CEBHA+), as part of the Research Networks for Health Innovation in Sub-Saharan Africa Funding Initiative of the German Federal Ministry of Education and Research. GT was supported by the South African Tuberculosis Bioinformatics Initiative (SATBBI), a Strategic Health Innovation Partnership grant from the South African Medical Research Council and South African Department of Science and Technology.

## Conflict of Interest Statement

The authors declare that the research was conducted in the absence of any commercial or financial relationships that could be construed as a potential conflict of interest.
