# Supplementary material for: Systematic Review of Genetic Factors in the Etiology of Esophageal Squamous Cell Carcinoma in African Populations
Source: Front Genet. 2019 Aug 2;10:642. doi: 10.3389/fgene.2019.00642 (PMC6687768; doi:10.3389/fgene.2019.00642)
Supplement: Supplementary file 1 [file Table_1.docx]

**Supplementary Table S1. Quality Assessment of Genetic Susceptibility Studies**

| **Study** | **Power calculations reported** | **Description of ESCC diagnosis** | **Screening of Controls for ESCC** | **Detailed population characteristics for cases** | **Detailed population characteristics for controls** | **Adjustments for population stratification** | **NCBI rs numbers** | **Assessment of HWE** | **Assessment of genotyping error** | **Reported data as risk ratios** | **Correction for multiple testing** | **Quality score (0 to 11)** |
| --- | --- | --- | --- | --- | --- | --- | --- | --- | --- | --- | --- | --- |
| Bye et al 2012 | Yes | Yes | No | Yes | Yes | No | Yes | Yes | No | Yes | Yes | 8 |
| Bye et al 2011 | Yes | Yes | No | Yes | No | No | Yes | Yes | No | Yes | Yes | 7 |
| Chen et al 2019 | Yes | Yes | No | Yes | Yes | No | Yes | Yes | Yes | Yes | Yes | 9 |
| Chelule at al 2006 | No | Yes | No | No | No | No | No | Yes | No | No | No | 2 |
| Dandara at al 2005 | No | Yes | No | No | No | No | No | No | No | Yes | No | 2 |
| Dandara at al 2006 | No | Yes | No | Yes | Yes | No | No | Yes | No | Yes | No | 5 |
| Dietzsch et al 2003 | No | Yes | No | Yes | Yes | No | No | No | No | Yes | No | 4 |
| Eltahir et al 2012 | No | Yes | No | No | No | No | No | No | No | Yes | No | 2 |
| Li et al 2005 | No | Yes | No | Yes | Yes | No | No | Yes | No | Yes | No | 5 |
| Li et al 2010 | Yes | Yes | No | Yes | Yes | No | Yes | Yes | No | Yes | Yes | 8 |
| Li et al 2008 | No | Yes | No | Yes | Yes | No | No | No | Yes | Yes | No | 5 |
| Matejcic et al 2011 | Yes | Yes | No | Yes | Yes | No | Yes | Yes | No | Yes | Yes | 8 |
| Matejcic 2015 | No | Yes | No | Yes | Yes | No | Yes | Yes | No | Yes | Yes | 7 |
| Strickland et al 2012 | No | Yes | Yes | Yes | No | No | Yes | Yes | No | No | No | 5 |
| Vogelsang et al 2012 | Yes | Yes | No | Yes | Yes | No | Yes | Yes | Yes | Yes | Yes | 9 |
| Vos et al 2003 | No | Yes | No | No | No | No | No | No | No | No | No | 1 |
| Zaahl et al 2005 | No | Yes | No | No | No | No | No | No | No | No | No | 1 |

ESCC, esophageal squamous cell carcinoma; HWE, Hardy Weinberg equilibrium; NCBI, National Center for Biotechnology Information (<https://www.ncbi.nlm.nih.gov/>): rs-numbers can be obtained from the NCBI dbSNP database, which is available at https://www.ncbi.nlm.nih.gov/snp/.
