# Supplementary material for: Systematic Review of Genetic Factors in the Etiology of Esophageal Squamous Cell Carcinoma in African Populations
Source: Front Genet. 2019 Aug 2;10:642. doi: 10.3389/fgene.2019.00642 (PMC6687768; doi:10.3389/fgene.2019.00642)
Supplement: Supplementary file 2 [file Table_2.docx]

**Supplementary Table S2. Quality Assessment of Somatic Variant Studies**

| **Study** | **Description of ESCC diagnosis** | **Tissues used: Cancerous & Normal neighbouring tissue, or blood** | **Detailed population characteristics** | **Variant classification and type** | **Confirmation of results** | **Amino acid change reported** | **Use of pathogenicity scoring described** | **Quality score (0 to 7)** |
| --- | --- | --- | --- | --- | --- | --- | --- | --- |
| Dietzsch et al 2003 | No | Yes | Yes | Yes | No | NA | NA | 3 |
| Dietzsch et al 2002 | Yes | Yes | Yes | Yes | No | NA | NA | 4 |
| Gamieldien et al 1998 | Yes | Yes | Yes | Yes | Yes | Yes | No | 6 |
| Liu et al 2016 | Yes | Yes | Yes | Yes | No | No | No | 4 |
| Naidoo et al 2005 | Yes | Yes | Yes | Yes | No | NA | NA | 4 |
| Patel et al 2011 | Yes | No | Yes | Yes | No | Yes | No | 4 |
| Victor et al 1990 | No | No | No | NA | NA | NA | NA | 0 |
| Vos et al 2003 | Yes | Yes | No | Yes | Yes | Yes | No | 5 |

ESCC, esophageal squamous cell carcinoma; NA, not applicable
