# Supplementary material for: Systematic Review of Genetic Factors in the Etiology of Esophageal Squamous Cell Carcinoma in African Populations
Source: Front Genet. 2019 Aug 2;10:642. doi: 10.3389/fgene.2019.00642 (PMC6687768; doi:10.3389/fgene.2019.00642)
Supplement: Supplementary file 3 [file Table_3.docx]

**Supplementary Table S3.** Summary of SNPs with r^2^>0.20.

| **Gene Symbol** | **Chromosome A** | **BP A^1^** | **SNP A** | **Chromosome B** | **BP B^1^** | **SNP B** | **R^2^** |
| --- | --- | --- | --- | --- | --- | --- | --- |
| *MSH2* | 2 | 47690411 | rs3771280 | 2 | 47709153 | rs10188090 | 0.787 |
| *CASP8* | 2 | 202097531 | rs3834129 | 2 | 202143928 | rs10931936 | 0.256 |
| *CASP8/* *ALS2CR12* | 2 | 202143928 | rs10931936 | 2 | 202162811 | rs13016963 | 0.315 |
| *ALS2CR12* | 2 | 202162811 | rs13016963 | 2 | 202202791 | rs10201587 | 0.409 |
| *CP* | 3 | 148919880 | rs35272481 | 3 | 148919962 | rs34237139 | 1 |
| *CP* | 3 | 148939861 | rs17838831 | 3 | 148939929 | rs34334174 | 0.515 |
| *CP* | 3 | 148939861 | rs17838831 | 3 | 148939933 | rs17838832 | 0.884 |
| *CP* | 3 | 148939861 | rs17838831 | 3 | 148940142 | rs17838834 | 0.884 |
| *CP* | 3 | 148939929 | rs34334174 | 3 | 148939933 | rs17838832 | 0.579 |
| *CP* | 3 | 148939929 | rs34334174 | 3 | 148940142 | rs17838834 | 0.580 |
| *CP* | 3 | 148939933 | rs17838832 | 3 | 148940142 | rs17838834 | 1 |
| *MSH3* | 5 | 79966029 | rs1805355 | 5 | 80008704 | rs1428030 | 0.809 |
| *NAT1* | 8 | 18080644 | rs1057126 | 8 | 18080651 | rs15561 | 0.908 |
| *PLCE1* | 10 | 96043732 | rs7084339 | 10 | 96058298 | rs3765524 | 0.970 |
| *PLCE1* | 10 | 96043732 | rs7084339 | 10 | 96066341 | rs2274223 | 0.572 |
| *PLCE1* | 10 | 96043732 | rs7084339 | 10 | 96068480 | rs11187850 | 0.224 |
| *PLCE1* | 10 | 96058298 | rs3765524 | 10 | 96066341 | rs2274223 | 0.585 |
| *PLCE1* | 10 | 96058298 | rs3765524 | 10 | 96068480 | rs11187850 | 0.233 |
| *PLCE1* | 10 | 96066341 | rs2274223 | 10 | 96068480 | rs11187850 | 0.349 |
| *ALDH2* | 12 | 112204427 | rs886205 | 12 | 112521448 | rs4767364 | 0.404 |
| *RUNX1* | 21 | 36357861 | rs2014300 | 21 | 36360884 | rs2834718 | 0.283 |
| *CHEK2* | 22 | 29115066 | rs4822983 | 22 | 29130300 | rs1033667 | 0.470 |

^1^Genome coordinate for the SNP
